# Non-structural Carbohydrate Metabolism in the Flesh of Stone Fruits of the Genus *Prunus* (Rosaceae) – A Review

**DOI:** 10.3389/fpls.2020.549921

**Published:** 2020-11-10

**Authors:** Robert P. Walker, Alberto Battistelli, Claudio Bonghi, María F. Drincovich, Rachele Falchi, María V. Lara, Stefano Moscatello, Giannina Vizzotto, Franco Famiani

**Affiliations:** ^1^Dipartimento di Scienze Agrarie, Alimentari e Ambientali, Università degli Studi di Perugia, Perugia, Italy; ^2^Istituto di Ricerca sugli Ecosistemi Terrestri, Consiglio Nazionale delle Ricerche, Porano, Italy; ^3^Department of Agronomy, Food, Natural Resources, Animals and Environment, University of Padova Agripolis, Legnaro, Italy; ^4^Facultad de Ciencias Bioquímicas y Farmacéuticas, Centro de Estudios Fotosintéticos y Bioquímicos, Consejo Nacional de Investigaciones Científicas y Técnicas, Universidad Nacional de Rosario, Rosario, Argentina; ^5^Department of Agricultural, Food, Environmental and Animal Sciences, University of Udine, Udine, Italy

**Keywords:** cell turgor regulation, fructans, invertases, primary metabolism, subcellular compartmentation, sugars, SPS, SuSy

## Abstract

Non-structural carbohydrates are abundant constituents of the ripe flesh of all stone fruits. The bulk of their content comprises sucrose, glucose, fructose and sorbitol. However, the abundance of each of these carbohydrates in the flesh differs between species, and also with its stage of development. In this article the import, subcellular compartmentation, contents, metabolism and functions of non-structural carbohydrates in the flesh of commercially cultivated stone fruits of the family *Rosaceae* are reviewed.

## Introduction

The term stone fruits refers to a number of species that are trees belonging to the genus *Prunus* of the rose family (*Rosaceae)*, that are characterized by fruits which possess a lignified endocarp called the stone or pit. These fruits are classified as drupes and are composed of a thin epicarp (skin), a fleshy mesocarp (flesh) and a woody/lignified endocarp which encloses the seed (Romani and Jennings, 1971). The commercially most important stone fruits are: plums (several species), among which the most important are the European plums (*P. domestica*) and the Asian or Japanese plums (*P. salicina*), sweet cherry (*P. avium*) and sour cherry (*P. cerasus*) (and hybrids between them), peach and nectarine (*P. persica*), apricot (*P. armeniaca*) and almond (*P. dulcis*). The rose family also includes other commercially important fruit trees and the main group is the pome fruits (subfamily *Pomoideae*), that have not been specifically considered in the present review. The term pome fruits refers to the fruit derived by the fusion of the ovary and receptacle (from which derives the flesh), and thus this is botanically a false fruit. Pome fruits include apple (*Malus domestica*), pear (*Pyrus communis*), quince (*Cydonia oblonga*), loquat (*Eriobotrya japonica*), medlar (*Mespilus germanica*), rowan (*Sorbus* spp.), and some other minor species.

The growth pattern, of either the whole fruit or the flesh of stone fruits, can usually be described as a double-sigmoidal curve, where three phases of growth are generally recognized: a first period of rapid growth defined as stage I, a second period with reduced growth depicted as stage II and a third period, characterized by a further rapid growth, defined as stage III (Lilleland, 1933; Pavel and DeJong, 1993b; Zuzunaga et al., 2001). The only exception is almond in which the flesh does not expand during stage III (Hawker and Buttrose, 1980). During stage I, all the components of the pericarp (epicarp, mesocarp and endocarp) increase greatly in size, and both the endocarp and the seed approach their maximum size. During stage II, the increase in the size of the flesh slows down and the endocarp, whose cells develop into sclerenchyma, hardens to form the stone. During stage III, there is a large increase in both the weight and volume of the fruit, arising from the expansion of the parenchyma cells of the flesh and skin, and it ripens. During ripening, the edible parts soften, change color and accumulate soluble sugars (Brady, 1993; Zuzunaga et al., 2001). In peach, some studies have subdivided stage I into stages Ia and Ib, and similarly stage III has been subdivided into stages III and IV (Chalmers and van den Ende, 1975; Scorza et al., 1991; Zanchin et al., 1994; Tonutti et al., 1997). However, because all stone fruit species are considered in the present review, we have used the traditional division into three stages.

**FIGURE 1:**
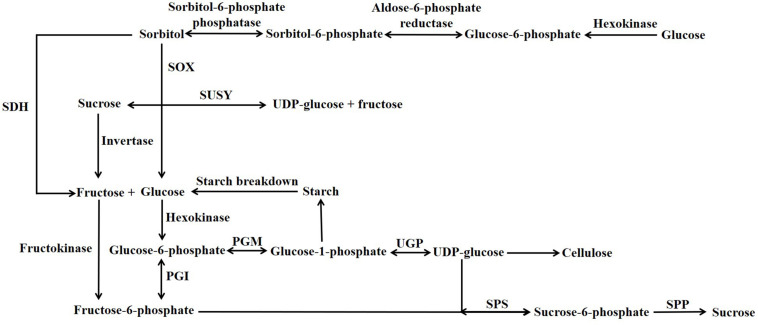
Simplified scheme illustrating sucrose and sorbitol degradation and synthesis. PGI, Phosphoglucose Isomerase; PGM, Phosphoglucomutase; SDH, Sorbitol Dehydrogenase; SOX, Sorbitol Oxidase; SPP, Sucrose phosphate phosphatase; SPS, Sucrose Phosphate Synthase; SUSY, Sucrose Synthase; UGP, UDP-Glucose Pyrophosphorylase.

A large number of different carbohydrates can be present in the flesh of stone fruits (Cirilli et al., 2016); however, in this article only abundant non-structural carbohydrates are considered, and their contents, metabolism and functions are reviewed. The bulk of the non-structural carbohydrates present in the flesh of all stone fruits at all stages of their development consists of one or more of the soluble sugars sucrose, glucose and fructose, the sugar alcohol sorbitol (henceforth referred to as a soluble sugar) and very small amounts of starch. The metabolism of these non-structural carbohydrates is linked by the sucrose cycle which, together with proteins that transport sugars across membranes, plays a pivotal role in determining the contents of these sugars in the different compartments of the cell. This cycle allows sugar utilization and accumulation to be coordinated and also plays a key role in maintaining the osmotic potential and turgor of different subcellular compartments (Li et al., 2012, 2016, 2018). A simplified scheme depicting the enzymes involved in the sucrose cycle and allied reactions is shown in Figure 1. Soluble sugars usually account for 70–90% of the dry weight of the ripe flesh and skin of commercially cultivated stone fruits (Moing et al., 1998; Baldicchi et al., 2015). The soluble solids content (SSC or °Brix) of the flesh and skin generally accounts for 9-22% of the fresh weight (Marshall, 1954; Moing et al., 1998; Famiani et al., 2012; Zanon et al., 2015; Baldicchi et al., 2015; Cirilli et al., 2016). A large percentage of the SSC usually consists of sugars, and in the ripe flesh of both peach (*P. persica*) and sweet cherry (*P. avium*) the percentage is typically 65-85% (Brady, 1993; Winkler and Knoche, 2018). However, there are exceptions, and in Japanese apricot (*P. mume*) this percentage is only about 16% (Otoguro and Kaneko, 1994). For most stone fruits, the content of sugars is a major determinant of the taste of the flesh and skin, and a high content is a major factor in determining the quality of the crop (Desnoues et al., 2014; Cirilli et al., 2016).

## Sorbitol and Sucrose Account for the Bulk of the Sugars Imported Into Stone Fruit Flesh

The bulk of the sugars required for stone fruit growth and development are imported into them, and only a very small amount is produced by the fruits’ own photosynthesis. These sugars largely arise from photosynthesis in the leaves during the same growing season that the fruit develops, although, during early fruit growth, some sugars arise from carbohydrates stored in the roots and crown during the previous season (Loescher et al., 1990; Pavel and DeJong, 1993a). Sucrose and sorbitol account for the bulk of sugars synthesized in peach leaves, and although sorbitol content is usually higher, the ratio of the contents of these two sugars is dependent on factors such as cultivar, rate of photosynthesis and age of the leaf (Escobar-Gutiérrez and Gaudillére, 1994; Moing et al., 1997; Hartman et al., 2017). Both sucrose and sorbitol are synthesized in the cytosol of mesophyll cells from either triose phosphate or the products of starch degradation (i.e., glucose and maltose) that are exported from the chloroplast. The triose phosphate and starch are produced by photosynthesis within the chloroplast (Cho et al., 2011). In the cytosol of mesophyll cells, a large proportion of triose phosphate, maltose and glucose are then converted to glucose-6-phosphate and fructose-6-phosphate and then used in both sucrose and sorbitol synthesis. Glucose-6-phosphate is used as a precursor for sorbitol synthesis (Figure 1). Aldose-6-phosphate reductase (also known as sorbitol-6-phosphate dehydrogenase) is a key regulatory enzyme utilized in sorbitol synthesis in leaves of Rosaceous plants (Figure 1; Hirai, 1981; Hartman et al., 2017; Shen C. et al., 2018). Sucrose synthesis in leaves predominantly utilizes the sucrose phosphate synthase (SPS) and not sucrose synthase (SuSy) pathway, and the SPS pathway uses as precursors both glucose-6-phosphate and fructose-6-phosphate (Figure 1). Sorbitol and sucrose then move to the phloem and enter it (phloem loading). It is a matter of debate as to the relative contributions of apoplastic (sugars enter apoplast before entering the phloem) and symplastic (sugars do not enter apoplast before entering the phloem) phloem loading in Rosaceous fruit trees such as peach and apple (Moing et al., 1997; Watari et al., 2004; Nadwodnik and Lohaus, 2008). Apoplastic loading in Rosaceous fruit trees, as in plants in which the process it has been studied in more detail, is likely to utilize Sugars Will Eventually be Exported Transporter (SWEETs: sucrose and hexose facilitator transporters) for the movement of at least sucrose into the apoplast, and sucrose transporters (SUC’s/SUTs: sucrose/H+ symporters) and sorbitol transporters for their uptake into the phloem (Watari et al., 2004; Jeena et al., 2019). Sorbitol transporters are encoded by a number of genes in Rosaceous fruit trees and the expression of each of these genes is often dependent on the tissue, stage of development and other factors (Gao et al., 2003; Li et al., 2015, 2018; Shen C. et al., 2018). Compared to sucrose transporters the role of these sorbitol transporters in phloem loading is less understood (Li et al., 2015; Shen C. et al., 2018). Whatever, the loading mechanism used, sugars then move to sink tissues by bulk flow through the phloem, and this is driven by a pressure gradient that is largely produced by an inflow of water into the phloem of source tissues by osmosis (Voitsekhovskaja et al., 2006). In the fruit sugars can then travel from the phloem to sink cells by either an intracellular (symplastic) route via plasmodesmata, an extracellular route (apoplastic) or a combination of both routes (Grappadelli et al., 2019). Both sucrose and sorbitol are transported in the phloem of stone fruits. The aphid stylet technique revealed the ratio of sorbitol to sucrose to be 2.8–4.5 in the phloem of both the leaf and shoot apex of peach (Moing et al., 1997; Nadwodnik and Lohaus, 2008). In total extracts of different organs of peach the ratio of sorbitol to sucrose differs, and this ratio is highest in the leaf lamina (about 2–3), medium in the fruit stalk (pedicel) (about 1.3–2) and lowest in the ripe fruit (about 0.03–0.2 in the flesh) (Table 1; Nii et al., 1994; Lo Bianco, 2009). In ripe peach flesh, more than 70% of soluble sugars is usually sucrose, and sorbitol content is commonly less than 15% (Moing et al., 1998). Why then is the sorbitol content of ripe peach flesh much less than that of sucrose? One potential explanation is that sucrose is the main sugar accumulated to make the fruit palatable/edible during ripening. Another explanation, not in contrast and potentially consequent to the first one, is that sorbitol and not sucrose is the main substrate utilized by metabolism (Desnoues et al., 2014). However, even if equal amounts of sorbitol and sucrose were imported into the flesh, the former would exceed the metabolic demands of the tissue. Thus, metabolic substrate in ripening peach flesh is required for both the synthesis of various components of the flesh and as a source for respiration (Famiani et al., 2016). However, most of the increase in the dry weight of peach flesh during stage III of development is the result of the increase in soluble sugar content, and in the flesh of fruits such as grape during the stage of development equivalent to stage III only about 9–14% of sugars present in the flesh are used in respiration (Famiani et al., 2014). An alternative explanation to why sorbitol content is low is that it is in part converted to sucrose, and there is evidence that this occurs. Firstly, feeding radiolabelled sorbitol to detached prune fruits (a European plum cultivar, i.e., *P. domestica*) via the pedicel resulted in 25–50% of the radiolabel in the flesh being present in sucrose. By contrast, when radiolabelled sucrose was fed to the fruits only 1.6% of the radiolabel was present in sorbitol (Hansen and Ryugo, 1979). Of course, in this latter study this does not preclude that there is a conversion of sorbitol to sucrose during its transfer from the pedicel to the parenchyma cells of the flesh. Secondly, key enzymes required for the synthesis of sucrose from sorbitol such as SPS, SuSy and/or sorbitol dehydrogenase are present in ripening peach flesh (Yamaki, 2010). This raises the question of which factors are important in determining the sorbitol content of the flesh. In contrast to peach flesh, the sorbitol content of cherry flesh can be quite high (Table 1), and it has been suggested that this is because imported sorbitol is little metabolized (Gao et al., 2003). In addition, in the flesh of some fruits such as pear and loquat, there is evidence that sorbitol can be re-synthesized; however, Desnoues et al. (2018) reported that this may not be the case in peach. Nevertheless, the potential re-synthesis of sorbitol in the flesh of stone fruits requires detailed investigation. In general, it seems that sorbitol is low in stone fruits which accumulate sucrose and tends to be higher in those that accumulate glucose/fructose (Table 1). On balance, it is not implausible that both imported sucrose and sorbitol are both quantitatively important metabolic substrates for stone fruit flesh, and that their relative contributions could depend on several factors such as species and developmental stage.

**TABLE 1:** Typical contents of soluble sugars (mg g^–1^ FW) in the flesh of unripe and ripe stone fruits.

	**Sorbitol**	**Sucrose**	**Fructose**	**Glucose**	
**Stage II – Unripe fruits**					
Apricot (common)	0.6	2	1	3	Baldicchi et al., 2015
Apricot (Japanese)	2.3	1.3	1.1	2.0	Otoguro and Kaneko, 1994
Cherry (sweet)		<1	9	23	Walker et al., 2011
Peach	3	3	13	14	Moriguchi et al., 1990
Plum (Japanese)	3	7	11	14	Famiani et al., 2012
					Donen, 1939
**Stage III – Ripe fruits**					
Apricot (common)	2.8	65	6	18	Baldicchi et al., 2015
Apricot (Japanese)	1.7	9.0	0.9	0.5	Otoguro and Kaneko, 1994
Cherry (sweet)	40	<1	65	75	Walker et al., 2011
					Ballistreri et al., 2013
	14	1.5	71	78	Winkler and Knoche, 2018
Cherry (sour)	16	4.2	43	52	Winkler and Knoche, 2018
Cherry (Morello) Line 16	43	low	60	68	Proietti et al., 2019
Cherry (Morello) Line 37	14	low	17	24	Proietti et al., 2019
Peach Hakuto	8	48	9	7	Moriguchi et al., 1990
Peach Pamirskii	9.3	115	11	6	Moing et al., 2003
Nectarine Summergrand	2.7	82	16	11	Moing et al., 2003
Plum (Japanese)	25	92	21	27	Famiani et al., 2012
					Donen, 1939
*Prunus davidiana*	0.3	10	3.8	2.7	Moing et al., 2003

## Content of Non-Structural Carbohydrates in the Flesh of Stone Fruits During Development

In the ripe flesh and skin of all stone fruits differing amounts of sucrose, glucose, fructose and sorbitol account for the bulk of the sugar content, and little or no starch is present (Table 1; Pavel and DeJong, 1993b; Moing et al., 1998; Desnoues et al., 2014, 2018; Baldicchi et al., 2015; Cirilli et al., 2016). However, the absolute amounts and relative proportions of each of these carbohydrates depends on the species of stone fruit and the cultivar, stage of development and growth conditions (Nonis et al., 2007; Morandi et al., 2008; Famiani et al., 2012; Desnoues et al., 2014; Baldicchi et al., 2015; Moscatello et al., 2019). In the ripe flesh of most, if not all, peaches the bulk of the soluble sugar content consists of sucrose, although smaller amounts of glucose, fructose and sorbitol are present. Further, there are differences among cultivars in the absolute amounts and relative proportions of these sugars (Moriguchi et al., 1990; Chapman and Horvat, 1990; Byrne et al., 1991; Chapman et al., 1991; Pavel and DeJong, 1993b; Vizzotto et al., 1996; Moing et al., 1998; Bae et al., 2014; Desnoues et al., 2014, 2018; Famiani et al., 2016; Cirilli et al., 2016). The amounts and proportions of sucrose, sorbitol, glucose and fructose present in the ripe flesh of most cultivars of common apricot (*P. armeniaca*) are similar to ripe peach flesh, however, differences have been detected between cultivars and tissues (i.e., peel and flesh) (Akin et al., 2008; Bae et al., 2014; Baldicchi et al., 2015; Xi et al., 2016). Nevertheless, peaches generally (but not always) contain more fructose than glucose, and the opposite has been observed in common apricot (Table 1; Cantín et al., 2009; Zanon et al., 2015; Baldicchi et al., 2015; Xi et al., 2016). The ripe flesh of both peaches and common apricots contains little starch (Pavel and DeJong, 1993b; Baldicchi et al., 2015). The soluble sugar content of the ripe flesh of Japanese apricots (*P. mume*) is low, and sucrose mostly accounts for this, although smaller amounts of glucose, fructose and sorbitol are present (Table 1; Otoguro and Kaneko, 1994). Glucose, fructose and sorbitol account for the bulk of the sugar content of the ripe flesh of all cherries studied; however, there is a variation in the proportions of these sugars between both species and cultivars (Table 1; Gao et al., 2003; Walker et al., 2011; Ballistreri et al., 2013; Winkler and Knoche, 2018; Proietti et al., 2019). The sucrose content of ripe flesh of different cherry species and their hybrids is usually very low, and generally less than 1% of the soluble sugar content (Walker et al., 2011; Ballistreri et al., 2013; Alrgei et al., 2016). However, certain genotypes can contain more, and one genotype contained around 4 mg g^–1^ FW (Alrgei et al., 2016). No starch was detected in the ripe flesh of either sweet cherry or sour cherry (*P. cerasus*) (Widdowson and McCance, 1935; Gao et al., 2003). Ripe plum flesh contains quite large amounts of sorbitol, sucrose, glucose and fructose (Table 1; Winkler and Knoche, 2018). However, there can be marked differences in the relative proportions of these between plum species and cultivars, and contents in the flesh and skin can be different (Donen, 1939; Forni et al., 1992; Nergiz and Yildiz, 1997; Singh et al., 2009; Famiani et al., 2012; Bae et al., 2014; Moscatello et al., 2019). No starch or very small amounts were detected in the ripe flesh of different plum species and several cultivars (*P. salicina*) (Widdowson and McCance, 1935; Donen, 1939; Moscatello et al., 2019). In the flesh of young fruits of different species of plum a low amount of starch (< 5 mg^−1^ DW) was present (Moscatello et al., 2019).

The absolute and relative abundance of the different sugars in the flesh of all stone fruit changes during development (Table 1; Gao et al., 2003; Walker et al., 2011; Famiani et al., 2012; Bae et al., 2014; Cirilli et al., 2016; Lombardo et al., 2011). In general, the content of glucose plus fructose in the flesh of peaches, plums and apricots is much higher than sucrose content during stages I and II. Then during stage III large amounts of sucrose accumulate and this becomes the most abundant soluble carbohydrate (Table 1). In common apricot it has been noted that sucrose imported into the flesh before stage III is mostly metabolized to other compounds and during stage III this is not the case (Zhang et al., 2017), and the data of Baldicchi et al. (2015) are consistent with this. This is not the situation in Japanese apricot flesh because low amounts of sucrose and other soluble sugars are present during stage III, and there is a large accumulation of citric acid (Otoguro and Kaneko, 1994). In peach, during stage III large amounts of sucrose accumulate, and glucose and fructose, expressed as g^–1^ FW, usually decrease, but the content per fruit increases (Moriguchi et al., 1990; Pavel and DeJong, 1993b; Vizzotto et al., 1996; Lo Bianco et al., 1999; Famiani et al., 2016). Both sorbitol and starch contents (in g^–1^ FW) are low throughout development (Moriguchi et al., 1990; Pavel and DeJong, 1993b; Vizzotto et al., 1996). Sorbitol content g^–1^ FW has been found to either increase or decrease during stage III (Moriguchi et al., 1990; Vizzotto et al., 1996; Lo Bianco et al., 1999; Lombardo et al., 2011). In the flesh and skin of peach starch is localized in chloroplasts (Zanchin et al., 1994). Starch content increases to a maximum of about 3 mg g^–1^ FW during mid stage I and then decreases to about 0–0.3 mg g^–1^ FW in ripening flesh (Lo Bianco et al., 1999; Lo Bianco and Rieger, 2002; Rodriguez et al., 2019). This is consistent with the development of the plastids in peach parenchyma cells; four weeks after full bloom the plastids of the parenchyma cells contain few thylakoids and no starch granules, however, one week later numerous starch grains are present and are localized in the plastids of the peripheral parts of the flesh (Masia et al., 1992; Zanchin et al., 1994). The appearance of starch occurs before the full development of the thylakoids, and therefore some starch could be synthesized from imported sugars (Zanchin et al., 1994). By the end of stage II, the plastids of the peripheral part of the flesh (unlike the inner part) possess well developed thylakoids and numerous starch grains. Thirteen weeks after full bloom (middle of stage III), these starch grains are absent, chloroplasts had been converted to chromoplasts and large intercellular spaces were present (Masia et al., 1992). Similar results were obtained in ‘Dixiland’ peach fruit during development (Rodriguez et al., 2019). The pattern of changes in carbohydrates content in apricot flesh during development is broadly similar to those described for peach (Baldicchi et al., 2015; Xi et al., 2016). In both sweet cherry and sour cherry, glucose and fructose are much more abundant than sucrose throughout development. During stage III large amounts of glucose and fructose are accumulated, and in many cultivars sorbitol is also accumulated (Table 1) (Gao et al., 2003; Walker et al., 2011; Ballistreri et al., 2013). In sour cherry flesh, starch was not detected at any stage of development (Gao et al., 2003). In the flesh of the Japanese plum Ozark Premier glucose and fructose are more abundant than sucrose before stage III, then the content g^–1^ FW of each of these sugars increases and sucrose becomes the most abundant (Table 1; Famiani et al., 2012). In the flesh of the Japanese plum Kelsey, somewhat similar observations were made, and changes in sorbitol content were generally similar to those of sucrose (Donen, 1939). Also in the flesh of Mirabolano (*P. cerasifera*), President (*P. domestica*) and Shiro (*P. salicina*), fructose and, especially, glucose are more abundant than sucrose during stages I and II, then, during stage III, sucrose becomes the most abundant (Moscatello et al., 2019). In the skin of Ozark Premier plum glucose and fructose were more abundant than sucrose before stage III, then the contents g^–1^ FW of these sugars increased and glucose and fructose remained more abundant than sucrose (Famiani et al., 2012). In plums, the content of sorbitol and its changes in content during development are dependent on the species/cultivar. In the flesh of Mirabolano, sorbitol was low during stage I and II of fruit development, then during stage III it increased greatly, and its abundance was similar to sucrose (Moscatello et al., 2019). In Shiro flesh sorbitol content was low and changed slightly during development, whereas in the flesh of President sorbitol content was low in young fruits and then increased to become the second most abundant sugar during stage III (Moscatello et al., 2019). As far as the hexoses are concerned, in the ripe flesh of six European plum cultivars, glucose was generally more abundant than fructose (Dugalic et al., 2014), and this was the case throughout the development of both the flesh and skin of ‘Ozark Premier’ plum (Famiani et al., 2012). This was also the case in the ripe flesh of Mirabolano, President and Shiro (Moscatello et al., 2019). However, in some varieties of Japanese plum fructose is more abundant than glucose (Singh et al., 2009). In the flesh of plums of Mirabolano, President and Shiro, glucose was the most abundant sugars throughout stages I and II, with amounts strongly dependent on the species: it was very high in Mirabolano, followed by President and Shiro (Moscatello et al., 2019).

## Subcellular Compartmentation of Non-structural Soluble Carbohydrates

In order to understand certain aspects of sugar metabolism in stone fruit flesh it is important to know the intracellular compartmentation of sucrose, glucose, fructose and sorbitol. In addition, it is clearly important to know what proportion of each of these sugars is located in the apoplast. However, determining these distributions is extremely difficult to do experimentally, and has not been established with certainty for any stone fruit (Desnoues et al., 2018). A modeling approach applied to peach flesh predicted an almost equal concentration of glucose, fructose and sorbitol in the cytosol and vacuole and a much higher concentration of sucrose in the vacuole than in the cytosol. Nevertheless, the bulk of the content of each of these sugars would be located in the vacuole because it occupies a very large fraction of cell volume (Desnoues et al., 2018). These results are at variance with the experimentally (the ‘wash out’ technique was used) derived results of Jiang et al. (2013), who found that a considerable proportion of the sugar content was in the cytoplasm. The results of Jiang et al. (2013) imply a very high cytoplasmic sugar concentration (because the cytoplasm only occupies a small proportion of the total cell volume) (Desnoues et al., 2018). Using the non-aqueous fractionation technique, Nadwodnik and Lohaus (2008) found that in mature peach leaves the bulk of the contents of sucrose, glucose, fructose and sorbitol were located in the vacuole. However, because of the large proportion of the cell occupied by the vacuole the concentrations of sorbitol and sucrose were higher in the cytosol, while the concentrations of glucose and fructose were much higher in the vacuole (Nadwodnik and Lohaus, 2008). Beshir et al. (2019) used the non-aqueous fractionation technique to determine the distribution of metabolites between different subcellular compartments during the development of apple flesh. The bulk of the contents of sucrose, glucose, fructose and sorbitol were located in the vacuole throughout development. These sugars were often also present in the cytosol and plastid, and this was dependent on both the sugar and stage of development. However, because of the large volume of the vacuole the actual concentration of some of these sugars could at certain stages of development be higher in the cytosol and plastid than in the vacuole (Beshir et al., 2019). It is plausible that in the flesh of stone fruits the subcellular distribution of sugars is comparable to apple flesh; however, it requires to be determined experimentally. In the flesh of stone fruit the glucose:fructose ratio usually is not one, and the reasons for this in peach have been recently considered. It was suggested that both the presence of isoforms of fructokinase with different affinities for fructose, and differences in the transport of fructose and glucose at the tonoplast could contribute to a higher content of glucose than fructose (Desnoues et al., 2014, 2018). However, the situation could be far more complicated than this because in tomato a glucose efflux transporter (a SWEET facilitator) located in the plasma membrane appears to be responsible for determining the ratio of glucose to fructose (Shammai et al., 2018). Thus, it is possible that different mechanisms are responsible for determining the ratio of glucose to fructose in the flesh of stone fruits, and which is predominant could depend on factors such as the species, cultivar and stage of development and environment.

The question arises as to what proportion of each sugar is located outside the cell. In grape berries, there is a large increase in the apoplastic concentrations of glucose and fructose just before the onset of ripening, and this higher concentration persists throughout ripening. During ripening, these hexoses are the predominant osmoticum in the apoplast with a total concentration of about 500 mM, and this is thought to be important in the process of turgor regulation associated with fruit softening (Wada et al., 2008). Similarly, in ripe sweet cherry flesh (and in the ripe flesh of sour cherry, European plum, tomato and a range of soft fruits) the most likely explanation for the low turgor pressure of the parenchyma cells is a build-up of apoplastic solutes (Knoche et al., 2014; Schumann et al., 2014). In kiwifruit, Gould et al. (2013) provided evidence that a large proportion of these apoplastic sugars arise not from apoplastic transport from the phloem, but from release from sink cells as part of a mechanism used to regulate cell turgor pressure. According to Schumann et al. (2014), in cherry the apoplastic volume is only around 10% of cell volume, and this would mean that the bulk of the sugar content was located within the cell. A physiological disorder of fruits termed watercore/glassines is associated with an increase in the content of apoplastic sorbitol (Gao et al., 2005).

## Metabolism of Non-structural Soluble Carbohydrates in the Flesh

Sucrose and sorbitol account for the bulk of the sugars imported into stone fruits; however, to enter metabolism they must be transformed into other compounds. For sucrose this usually requires either invertase (which catalyzes: sucrose + H_2_O → glucose + fructose) or sucrose synthase (SuSy, which catalyzes: sucrose + UDP ↔ UDP-glucose + fructose) (Figure 1). On the basis of the pH optimum for their catalytic activity, invertases are subdivided into the acid and neutral/alkaline invertases (henceforth referred to as neutral invertase). Acid invertases are located in either the vacuole or cell wall; whereas, the neutral invertases are located in either the cytosol, nucleus, mitochondrion or plastid (Kingston-Smith et al., 1999; Sturm, 1999; Nonis et al., 2008; Vargas and Salerno, 2010). SuSy is often located in the cytosol, but it is not uncommonly found in other locations (Granot and Stein, 2019). In plants (including those stone fruits studied), acid invertase, neutral invertase and SuSy are each encoded by small gene families (Nonis et al., 2008; Zhang et al., 2015; Vimolmangkang et al., 2016; Wang et al., 2017; Shen L. B. et al., 2018; Granot and Stein, 2019). The genes for the invertases, SuSy, SPS and SDH present in the peach genome are shown in Table 2. Amongst these, a SuSy (Prupe.5G241700.1) is a candidate gene for controlling the SSC because it is within the interval (located between 12 and 18 Mbp of chromosome 5) of a Quantitative Trait Loci linked to SSC (Nuñez-Lillo et al., 2019; Rawandoozi et al., 2020).

**TABLE 2:** Genes encoding for the invertases, SuSy, SPS and SDH identified in the peach genome.

**Transcript ID version 2**	**Transcript ID version 1**	**Enzyme**	**TAIR match**	**Location**	**References transcripts analysis**
**Sucrose synthase**					
Prupe.1G131700.1	ppa001573m, ppa001845m	Sucrose synthase	ATSUS2	Pp01:10355300-10373098	Zhang et al., 2015; Vimolmangkang et al., 2016; Aslam et al., 2019
Prupe.1G192300.1		Sucrose synthase	n.a.	Pp01: 17838341-17839225	Aslam et al., 2019
Prupe.2G242300.1	ppa001535m	Sucrose synthase	ATSUS3	Pp02:26135523-26137574	Zhang et al., 2015; Vimolmangkang et al., 2016
Prupe.3G014100.1	ppa017606m	Sucrose synthase	ATSUS6	Pp03:1010886-1015717	Zhang et al., 2015; Vimolmangkang et al., 2016; Aslam et al., 2019
Prupe.5G241700.1	ppa001135m	Sucrose synthase	ATSUS6	Pp05:18195911-18200676	Zhang et al., 2015; Vimolmangkang et al., 2016; Aslam et al., 2019
Prupe.7G192300.1	ppa001535m	Sucrose synthase	ATSUS4	Pp07:18350215-18356360	Lombardo et al., 2011 (SS); Zhang et al., 2015; Vimolmangkang et al., 2016
Prupe.8G264300.1	ppa002723m	Sucrose synthase	ATSUS3	Pp08:22179197-22184773	Zhang et al., 2015; Vimolmangkang et al., 2016; Aslam et al., 2019
**Invertase**					
Prupe.1G111800.1	ppa002847m	Alkaline/neutral invertase	Plant neutral invertase family protein	Pp01:8933938-8938072	Lombardo et al., 2011 (NI1); Vimolmangkang et al., 2016; Aslam et al., 2019
Prupe.1G365400.1	ppa025225m	Alkaline/neutral invertase	Plant neutral invertase family protein	Pp01:33537122-33539832	Vimolmangkang et al., 2016
Prupe.1G556900.1		Cytosolic invertase	CINV2		Aslam et al., 2019
Prupe.2G075000.1	ppa004112m	Alkaline/neutral invertase	Plant neutral invertase family protein	Pp02:11444746-11449675	Vimolmangkang et al., 2016
Prupe.2G083900.1	ppa002625m	Alkaline/neutral invertase	alkaline/neutral invertase	Pp02:13283820-13288728	Lombardo et al., 2011 (NI2); Vimolmangkang et al., 2016
Prupe.2G191400.1	ppa019684m	Cytosolic invertase	S CINV2	Pp02:23073519-23078158	Vimolmangkang et al., 2016
Prupe.2G277900.1	ppa002732m	Vacuolar invertase	ATBETAFRUCT4, VAC-INV	Pp02:27860807-27865049	Vimolmangkang et al., 2016
Prupe.3G009500.1	ppa003412m	Cell wall invertase	ATCWINV1, ATBFRUCT1	Pp03:606163-610062	Vimolmangkang et al., 2016
Prupe.3G048300.1	ppa003470m	Cell wall invertase	ATCWINV1, ATBFRUCT1	Pp03:3405921-3409831	Vimolmangkang et al., 2016; Aslam et al., 2019
Prupe.5G075600.1	ppa002334m	Vacuolar invertase	ATBETAFRUCT4, VAC-INV	Pp05:8955357-8959521	Vimolmangkang et al., 2016; Aslam et al., 2019
Prupe.6G122600.1	ppa002614m	Alkaline/neutral invertase	INV-E, At-A/N-InvE	Pp06:9128385-9135532	Vimolmangkang et al., 2016
Prupe.6G309800.1	ppa002385m	Alkaline/neutral invertase	Plant neutral invertase family protein	Pp06:27815656-27819789	Lombardo et al., 2011 (NI3); Vimolmangkang et al., 2016; Aslam et al., 2019
Prupe.7G103100.1	ppa022745m	Cell wall invertase	ATCWINV4, CWINV4	Pp07:13274130-13276372	Vimolmangkang et al., 2016
Prupe.7G103200.1	ppa019728m	Cell wall invertase	ATCWINV4, CWINV4	Pp07:13285991-13289044	Vimolmangkang et al., 2016; Aslam et al., 2019
Prupe.7G103300.1	ppa003343m	Cell wall invertase	ATCWINV2, CWINV2	Pp07:13293841-13296921	Lombardo et al., 2011 (AI2); Vimolmangkang et al., 2016
Prupe.7G103400.1	ppa004218m	Cell wall invertase	ATCWINV4, CWINV4	Pp07:13299771-13304593	Vimolmangkang et al., 2016
Prupe.8G159800.1	ppa003483m	Alkaline/neutral invertase	Plant neutral invertase family protein	Pp08:16872814-16878303	Vimolmangkang et al., 2016; Aslam et al., 2019
**Sucrose-phosphatase synthase**					
Prupe.1G159700.1	ppa000622m	Sucrose-phosphatase synthase	ATSPS3F, SPS3F	Pp01:12702147-12709381	Vimolmangkang et al., 2016; Aslam et al., 2019
Prupe.1G483200.1	ppa000636m	Sucrose-phosphatase synthase	ATSPS1F, SPS1F	Pp01:40288494-40295210	Lombardo et al., 2011 (SPS2); Vimolmangkang et al., 2016; Aslam et al., 2019
Prupe.7G249900.1	ppa000639m	Sucrose-phosphatase synthase	ATSPS1F, SPS1F	Pp07:21151882-21157785	Lombardo et al., 2011 (SPS1); Vimolmangkang et al., 2016; Aslam et al., 2019
Prupe.8G003700.1	ppa000716m	Sucrose-phosphatase synthase	ATSPS4F	Pp08:302873-308259	Vimolmangkang et al., 2016; Aslam et al., 2019
**Sorbitol dehydrogenase**					
Prupe.1G057900	ppa007458m	Sorbitol dehydrogenase	GroES-like zinc-binding alcohol dehydrogenase family protein	Pp01:4111842-4114761	
Prupe.2G288800	ppa007458m	Sorbitol dehydrogenase	GroES-like zinc-binding alcohol dehydrogenase family protein	Pp02:28363713-28365855	Lombardo et al., 2011 (SDH1)
Prupe.4G240300	ppa007327m	Sorbitol dehydrogenase	GroES-like zinc-binding alcohol dehydrogenase family protein	Pp04:15817263-15819830	
Prupe.8G142900	ppa007374m	Sorbitol dehydrogenase	GroES-like zinc-binding alcohol dehydrogenase family protein	Pp08:15994437-15996971	
Prupe.8G143000	ppa007343m	Sorbitol dehydrogenase	GroES-like zinc-binding alcohol dehydrogenase family protein	Pp08:15999040-16001622	

*In the table are reported the IDs assigned to each gene in the first (The International Peach Genome Initiative – Verde et al., 2013) and the second version (Verde et al., 2017) of the peach genome to make easier the identification of genes for which have been carried out the analysis of transcripts (see references in the last column). For each gene is also reported the location in the peach genome (to retrieve all the information see at Prunus persica Genome v2.0.a1: JBrowse | Genome page) and best hits against the Arabidopsis genome sequences (TAIR, https://www.arabidopsis.org/Blast/).*

In the flesh of both stone fruits and pome fruits, both sucrose and sorbitol can be broken down and then potentially resynthesized as either sucrose or sorbitol, and this turnover is referred to as the sucrose cycle (Li et al., 2012, 2016, 2018). A simplified scheme depicting the enzymes utilized in this process is shown in Figure 1. This cycle is central to carbohydrate metabolism in sink tissues and in conjunction with proteins that transport sugars across membranes allows sugar utilization and accumulation to be coordinated. In addition, the cycle together with sugar transporters plays a key role in maintaining the osmotic potential and turgor of different subcellular compartments (Li et al., 2012, 2016, 2018). There is a large increase in the content of soluble sugars g^–1^ DW of flesh during stage III of development in stone fruits such as peach (Pavel and DeJong, 1993b; Moing et al., 1998). This shows that after the onset of ripening a much lower proportion of imported sugars are used in the synthesis of compounds other than non-structural carbohydrates than before this time. In stone fruits the amount of CO_2_ released g^–1^ FW of flesh is much lower during stage III as compared to stage I (Pavel and DeJong, 1993a; Famiani et al., 2016). The bulk of this CO_2_ arises from the action of the Krebs cycle which is associated with the respiratory processes that provide ATP and reductant. However, the reason for this lower CO_2_ output during stage III likely arises from a dilution effect brought about by a large increases in the ratio of the vacuole to cytoplasm of fruit parenchyma cells (Famiani et al., 2016, 2020). In stone fruits the relative importance of enzymes of the sucrose cycle (and enzymes catalyzing allied reactions), sugar transporters and other factors in determining the increased accumulation of sugars during ripening is unknown. In apple, detailed studies have provided a useful model of sugar metabolism in the flesh at different stages of development (Li et al., 2018). The reader is referred to this work because it provides valuable insights into the situation in stone fruits.

### Membrane Sugar Transporters

The proteins that transport sugars across membranes in plants can be divided into three families: sucrose transporters (SUC’s/SUTs: sucrose/H^+^ symporters), monosaccharide transporters (MST’s: hexose/H^+^ antiporters/symporters) and Sugars Will Eventually be Exported Transporter (SWEETs: sucrose and hexose facilitator transporters). At least some sorbitol transporters are members of the MST family (Cheng et al., 2018; Ma et al., 2018). In stone fruits, there have been few detailed studies of these transporters. The abundance and locations of transcripts encoding SUTs have been investigated in peach flesh, and possible roles in sucrose retrieval from the apoplast and in sucrose release from the vacuole suggested (Zanon et al., 2015). In peach flesh two SWEET genes were expressed, and their protein products might play a role in the unloading of sucrose from the phloem (Zanon et al., 2015). Earlier uptake studies in peach flesh are consistent with a proportion of apoplastic sucrose being hydrolysed by cell wall invertase, and the hexoses produced being transported into parenchyma cells by a hexose transporter(s) (Vizzotto et al., 1996). A recent study of a tonoplast sugar transporter (*PpTST1*; a MST) found that the gene for this transporter is located in a quantitative trait locus (QTL) for sucrose, and that transient silencing of *PpTST1* significantly reduced sucrose accumulation (Peng et al., 2020). The abundance of transcripts of two sorbitol/proton symporters in sour cherry flesh are consistent with a proportion of sorbitol being unloaded from the phloem into the apoplast (Gao et al., 2003).

### Sucrose Synthase (SuSy) and Sucrose Phosphate Synthase (SPS)

Sucrose synthase activity is present in extracts of peach flesh throughout its development, and the amounts of activity in peach flesh and in some other fruits are shown in Table 3. In peach flesh, SuSy activity g^–1^ FW is highest during the earlier part of stage I, it then declines, and then, according to some studies, increases during ripening (Moriguchi et al., 1990; Hubbard et al., 1991) or, to others, does not (Vizzotto et al., 1996; Lo Bianco and Rieger, 2002). A survey of a large number of peach genotypes showed that there are considerable differences in the amounts of SuSy present in the flesh of the different genotypes (Table 3; Desnoues et al., 2014). Similarly, there are differences in the abundance of SuSy RNA transcripts among cultivars of peach (Vimolmangkang et al., 2016). Peach contains six SuSy genes (Verde et al., 2013) and transcripts arising from three of these are abundant in the flesh, and thus the function of SuSy in peach fruits is complex, not well understood, and could depend on both the tissue and its stage of development (Zhang et al., 2015). In apple, the situation appears to be similar (Tong et al., 2018). From studies of other plants, it is clear that SuSy could potentially play diverse roles in sucrose metabolism in stone fruits, and these include phloem/xylem metabolism, cellulose and callose synthesis, and the provision of substrate for metabolism especially when O_2_-supply is low (Granot and Stein, 2019). Nevertheless, the absolute requirement for SuSy in some of these processes has been questioned (Barratt et al., 2009).

**TABLE 3:** Typical approximate activities of sucrose synthase and invertases (μmol g^–1^ FW h^–1^) in the flesh of fruits at the stage of development roughly equivalent to late stage I in stone fruits (young fruits) and during ripening.

	**Sucrose synthase (cleavage)**	**Neutral invertase**	**Total acid invertase**	
**Late stage I – Young fruits**				
Cherry (sweet)			22	Krishnan and Pueppke, 1990
Peach	10–20	2–12	6–30	Moriguchi et al., 1990
				Vizzotto et al., 1996
Kiwifruit	40	3	8	Moscatello et al., 2011
Grape (hexose accumulator)	0.15–5		130–200	Takayanagi and Yokotsuka, 1997; Wu et al., 2011
Grape (sucrose containing)	0.15–2		< 4	
Tomato (hexose accumulator)	30		240	Yelle et al., 1991
Tomato (sucrose accumulator)	18		4	
Asian pear	2–10		8–33	Moriguchi et al., 1992
Strawberry	6	2.5	25	Hubbard et al., 1991
Grapefruit juice sacs	15	3.9	25	Lowell et al., 1989
Major vascular bundles	12	2.0	49	
Albedo of peel	4	1.6	91	
**Stage III – Ripening**				
Cherry (sweet)			180	Krishnan and Pueppke, 1990
Peach	1–14	0–1.5	2–6	Moriguchi et al., 1990
				Vizzotto et al., 1996
Peach	5–25	< 3	0	Hubbard et al., 1991
Peach (large number of genotypes)		average		Desnoues et al., 2014
	3.6	1.8	1.5	
		range		
	0–13	0–11	0–6	
Kiwifruit	4	1	4	Moscatello et al., 2011
Grape (hexose accumulator)	0.3–5		150–250	Takayanagi and Yokotsuka, 1997; Wu et al., 2011
Grape (sucrose containing)	0.3–5		< 1.5	
Tomato (hexose accumulator)	1–3		1200	Yelle et al., 1991
Tomato (sucrose accumulator	0.06–2.5		< 0.3	
Strawberry	6	10	3	Hubbard et al., 1991
Asian pear	1–6		1–7	Moriguchi et al., 1992
Grapefruit				Lowell et al., 1989
Juice sacs	0.1	0.6	0.4	
Major vascular bundles	1.0	0.3	1.3	
Albedo of peel	0.1	0.5	1.9	

SuSy catalyzes a reversible reaction, and Moriguchi and Yamaki (1988) and Moriguchi et al. (1990) suggested that in ripening peach flesh SuSy could play a role in sucrose synthesis. In plants either SPS or SuSy are required for the synthesis of sucrose from either glucose or fructose; however, the predominant route in most tissues is via SPS (Hubbard et al., 1991). The activity of SPS (which catalyses: fructose-6-phosphate + UDP-glucose ↔ sucrose-6-phosphate + UDP – Figure 1) has been measured in peach flesh. Thus, Vizzotto et al. (1996) found that throughout development SPS activity was about 2.5 μmol g^–1^ FW h^–1^, whilst Moriguchi et al. (1990) reported values of about 1 μmol g^–1^ FW h^–1^. In contrast, Hubbard et al. (1991) found that during the ripening of peach flesh SPS activity increased from about 8 to 14 μmol g^–1^ FW h^–1^ and SuSy activity increased from 5 to 25 μmol g^–1^ FW h^–1^, however, they were non-committal as to whether SuSy functioned in sucrose synthesis. A study carried out on different peach genotypes showed that there were considerable differences in the amounts of SPS present in their flesh; with an average value of 0.6 and a range of 0–2.7 μmol g^–1^ FW h^–1^ (Desnoues et al., 2014). Similarly, the abundance of transcripts arising from different SPS genes differs among cultivars of peach (Vimolmangkang et al., 2016). The expression of the different members of the gene families that encode SuSy and SPS (4 genes, according to Verde et al., 2013) has been investigated in peach flesh, and only certain member(s) of each family show increased expression during ripening (Lombardo et al., 2011; Zhang et al., 2015; Vimolmangkang et al., 2016). Yamaki (2010) reported that SuSy can function in both the synthesis of sucrose and its degradation in the flesh of fruits and was of the opinion that this was dependent on the species of fruit, tissue, stage of development and isoform of SuSy.

### Invertases

Although the acid and neutral invertases have different pH optima these are quite broad, therefore, when an enzyme activity assay is conducted at either acidic or alkaline pH, both invertases can contribute to the total activity that is measured (Lowell et al., 1989; Burger and Schaffer, 2007). Hence, for tissues such as cherry flesh that contain high amounts of acid invertase (Table 3), in crude extracts it is impossible to obtain reliable measurements of the neutral invertase activity. On the other hand, peach flesh contains much lower amounts of acid invertase, and it has been possible to measure both acid and neutral invertase in crude extracts (Vizzotto et al., 1996). In tissues, in which one form of invertase is much more abundant than the other, it is possible to rapidly separate them by using Concanavalin A chromatography (Walker et al., 1997).

A comparison between neutral invertase activity in extracts of peach flesh and in some other fruits are shown in Table 3. Neutral invertase is present in peach flesh throughout its development, and its activity shows two peaks: one occurring during the first part of stage I and the other during stage II (Vizzotto et al., 1996). By contrast, the amount of neutral invertase protein visualized on western blots (loaded so that each track contained an equal amount of total protein) was highest in ‘Redhaven’ peach flesh during stage II (Nonis et al., 2007). This difference is likely a result of the decline in total protein g^–1^ flesh FW during stage I (Lombardo et al., 2011; Famiani et al., 2012). In ‘Springcrest’ peach flesh, neutral invertase protein declined much more during development than in ‘Redhaven’ peach flesh (Nonis et al., 2007). Considerable differences in the amounts of neutral invertase were pointed out in the flesh of peach genotypes (Table 3; Desnoues et al., 2014). In the flesh of ‘Dixiland’ peach, neutral invertase activity per mg of total protein was lower during stage I, and was much higher during stages II to IV (Lombardo et al., 2011). In both peach and grape, neutral invertase is encoded by a small gene family, and in peach eight genes have been identified, six of which are expressed in the fruit (Nonis et al., 2008; Vimolmangkang et al., 2016). Indeed, in peach the abundance of transcripts arising from each of these genes is dependent on both the stage of development and the cultivar (Vimolmangkang et al., 2016). Analysis of the neutral invertase gene family in different plant species shows that they can be divided into α (located in organelles) and β (located in the cytosol) neutral invertases. These two forms of neutral invertase are of ancient origin (Nonis et al., 2008; Shen L. B. et al., 2018). In peach flesh, as in a range of other tissues, cytosolic neutral invertase is likely to play a role in providing substrates for metabolism (Ricardo and ap Rees, 1970; Nonis et al., 2007; Barratt et al., 2009; Borsani et al., 2009; Rossouw et al., 2010). Other potential functions for the neutral invertases in plants include osmoregulation, signaling and the involvement in responses to various stresses (Nonis et al., 2007; Dahro et al., 2016).

Acid invertase activity is present in the flesh of stone fruits, and the amounts of its activity in these and some other fruits is shown in Table 3. A major difficulty arises in determining whether the acid invertase activity in extracts of a tissue is due to the vacuolar or cell wall enzyme. Usually, the acid invertase activity is measured in both the soluble and particulate fractions obtained after centrifugation of extracts. However, there can be considerable uncertainty as to whether during extraction cell wall invertase was solubilised or vacuolar invertase became bound to the cell wall fraction (Ricardo and ap Rees, 1970; Lowell et al., 1989). In tissues, such as the flesh of grape and cherry, in which vacuolar acid invertase is very abundant (Table 3), this problem regarding cell wall invertase can be particularly acute. Indeed, in grape flesh attempts to determine the relative proportions of vacuolar and cell wall invertase activity has been the subject of several studies, and these proportions remain uncertain (Davies et al., 2012). On the basis of these pieces of evidence, at the moment, it appears evident that it is not possible to determine the single contribution of the two forms (vacuolar and cell wall) to the acid invertase activity when the latter is measured in crude extracts of soluble proteins. To determine this further investigation is required. Vacuolar invertases have a low pI (isoelectric point) whereas cell wall invertases have a high pI (Sturm, 1999). The pI determination of the invertases present in soluble extracts of a tissue by chromatofocusing, as well as the use of antibodies specific for either the vacuolar or cell wall acid invertase (Simpson et al., 1991; Tang et al., 1999; Famiani et al., 2000) will give a strong indication of their subcellular location.

Soluble acid invertase activity is present in peach flesh throughout development, and its activity g^–1^ FW is highest during the early part of stage I, then it decreases, and during stage III one study found that it increased whilst another did not (Moriguchi et al., 1990; Vizzotto et al., 1996). However, there are considerable differences in the amounts of soluble acid invertase present in the flesh of different peach genotypes (Table 3; Desnoues et al., 2014). Soluble acid invertase activity per mg of total protein showed a different pattern of changes during development and was lowest during stage I (Lombardo et al., 2011). Insoluble acid invertase is present in peach flesh throughout development and its activity g^–1^ FW is highest in early stage I and then it declines, and, according to one study, it increases during stage II whilst another study found that this increase occurred during stage III (Moriguchi et al., 1990; Vizzotto et al., 1996). Further, it was found that acid invertase activity during stage III was largely in the insoluble fraction, and it was suggested that the enzyme was predominantly located in cell wall (Ugalde et al., 1988; Moriguchi et al., 1990, 1991). In the peach genome, two genes for vacuolar and six genes for cell wall acid invertase have been identified (Vimolmangkang et al., 2016), and this is similar to the acid invertase gene family from some other plants (Wang et al., 2017). In peach flesh, there are differences among cultivars in the abundance of transcripts arising from the different acid invertase genes, and the abundance of these transcripts also changes during development (Vimolmangkang et al., 2016). These differences among cultivars are likely related to the different amounts of acid invertase activity present in the flesh of different peach genotypes (Table 3; Desnoues et al., 2014). In the flesh of stone fruits, as in a range of other tissues, the acid invertases could potentially function in the provision of substrate for metabolism, osmoregulation associated with the regulation of turgor pressure, phloem unloading and the generation of hexoses used in sugar sensing (Sturm, 1999; Sturm and Tang, 1999). These roles are discussed in more detail in the section dealing with functions of sugars.

In many sink tissues (such as grape berry flesh, tomato fruit flesh, sugarcane internode, sugar beet roots and carrot tap roots) there is an inverse relationship between sucrose content and the abundance of vacuolar invertase (Ricardo and ap Rees, 1970; Sturm and Tang, 1999). In ripening cherry flesh the activity of soluble acid invertase is 30–90 times higher than in ripening peach flesh (Krishnan and Pueppke, 1990; Table 3). Cherry flesh unlike that of peach contains little sucrose and large amounts of glucose and fructose (Table 1). It is therefore likely that the abundance of vacuolar acid invertase in the flesh of stone fruits is a major factor in determining their sucrose:(glucose + fructose) ratio (Walker et al., 2011). However, in some tissues it has been found that the relationship between the ratio of sucrose:(glucose + fructose) and the abundance of soluble invertase is not linear (Zhu et al., 1997; Beauvoit et al., 2014). Thus, at lower sucrose content and higher glucose plus fructose content more acid invertase activity is required to bring about the same decrease in sucrose content than is required at high sucrose content and lower glucose plus fructose content. One explanation for this is that *in vivo* vacuolar acid invertase activity is markedly inhibited by high concentrations of these hexoses (Walker and Pollock, 1993; Walker et al., 1997; Kingston-Smith et al., 1999), and work on tomato fruit supports this explanation (Beauvoit et al., 2014). Desnoues et al. (2014) failed to find a correlation between soluble acid invertase activity and sucrose content in the flesh of a range of peach genotypes; however, the proportions of invertase activity measured that were due to the vacuolar and cell wall forms of the enzyme were not determined.

### Enzymes of Sorbitol Metabolism

In order to enter metabolism imported sorbitol must be transformed into other compounds. To achieve this, potentially either NAD-sorbitol dehydrogenase (NAD-SDH) (NAD-SDH catalyzes: sorbitol + NAD^+^ ↔ fructose + NADH + H^+^), NADP-sorbitol dehydrogenase (NADP-SDH) or sorbitol oxidase (SOX) can be used (Figure 1) (Loescher, 1987; Lo Bianco and Rieger, 2002; Yamaki, 2010; Wang et al., 2016). In peach fruits, the relative contributions of NAD-SDH, NADP-SDH and SOX to the catabolism of sorbitol are dependent on both the tissue and its stage of development (Moriguchi et al., 1990; Lo Bianco et al., 1999; Lo Bianco and Rieger, 2002; Moscatello et al., 2017). Yamada et al. (2001) stated that in some earlier studies of NAD-SDH activity in peach flesh unsuitable extraction/assay conditions were used which led to inaccurate measurements of its abundance. Thus, although Moriguchi et al. (1990) detected SOX in peach flesh throughout its development, NAD-SDH and NADP-SDH were barely detectable. In the flesh of Encore peach NAD-SDH was only detected during stage III and SOX was not detected during at least stages I and II (Lo Bianco et al., 1999). However, later studies showed that in Encore peach not too dissimilar amounts of both NAD-SDH and SOX specific activity were detected in the flesh during stages I and III (Lo Bianco and Rieger, 2002). Similarly, Yamada et al. (2001) showed that both SDH protein and activity g^–1^ FW were highest early in development, then declined and subsequently increased during ripening. In sour cherry flesh, only very low activities of NAD-SDH were present throughout development and SOX was not detected (Gao et al., 2003). In Japanese plum flesh both SDH and SOX were present, and their specific activities (expressed on a FW basis) decreased during development (Kim et al., 2015; Farcuh et al., 2017). Nevertheless, some very different values for both NAD-SDH and SOX are reported for peach flesh at comparable stages of development. Thus, in peach flesh NAD-SDH activity for stages I-III were 1.3, 0.1 and 0.2 μmol g^–1^ FW h^–1^. The values for SOX at these stages of development were 0.9, 0 and 0.3 μmol g^–1^ FW h^–1^, respectively (Lo Bianco and Rieger, 2002). By contrast, for peach flesh during stage III, Morandi et al. (2008) reported values of SOX activity of 10–11.4 μmol g^–1^ FW h^–1^ and for NAD-SDH activity of 0.07–0.09 μmol g^–1^ FW h^–1^. Yamada et al. (2001) reported values of NAD-SDH activity in peach flesh at stages I-III of 420, 30 and 240 μmol g^–1^ FW h^–1^, respectively. In the ripening flesh of a large number of peach genotypes, the average value of NAD-SDH was 2.4 μmol g^–1^ FW h^–1^ and the range was 0–18 μmol g^–1^ FW h^–1^ (Desnoues et al., 2014). In the ripe flesh of 4 cultivars of peach the activity of NAD-SDH was 0.06–0.27 μmol g^–1^ FW h^–1^ (Kanayama et al., 2005). Moriguchi et al. (1990) reported values of SOX activity in peach flesh at stages I-III of 0.08, 0.03 and 0.03 μmol g^–1^ FW h^–1^, respectively. In the ripening flesh of a large number of peach genotypes, the average value of SOX was 2.4 μmol g^–1^ FW h^–1^ and the range was 0–11 μmol g^–1^ FW h^–1^ (Desnoues et al., 2014). In peach endocarp at stage I the activities of SOX was 2.3 and NAD-SDH was 0.07 μmol g^–1^ FW h^–1^ (Lo Bianco and Rieger, 2002). Clearly, in order to further understand the function of NAD-SDH and SOX in stone fruits it is essential to determine the abundance of the different forms of these enzymes in the various tissues of the fruit and seed. In the peach genome there are seven genes that encode NAD-SDH and at least four are expressed in the fruit (Cirilli et al., 2016). Apple contains nine NAD-SDH genes and five of these are expressed in the fruit plus seed (Nosarzewski and Archbold, 2007).

Much less work has been done on the enzymes involved in the catabolism of sucrose and sorbitol in plums and apricots than in peach. The studies on plum (Kim et al., 2015; Farcuh et al., 2017) and apricot (Xi et al., 2016) were not inconsistent with the results obtained for peach and cherry in terms of which enzymes were present and the patterns of changes in their activity during development. However, in the case of Japanese plum, the amounts of enzyme activity were orders of magnitude higher (Kim et al., 2015; Farcuh et al., 2017), and the reason for this difference requires clarification.

## Fructans in the Flesh of Stone Fruits

Low amounts of fructan have been detected in the ripe flesh of one peach cultivar (4 mg g^–1^ FW) (Muir et al., 2007) and ripe plum flesh (c0.6 mg g^–1^ FW) (L’homme et al., 2001). Fructans are fructose oligomers/polymers, and the bulk of their content is usually located in the vacuole. In plants that accumulate large amounts of fructan they are synthesized by a range of fructosyl transferase enzymes, which have evolved from the vacuolar acid invertases (Kingston-Smith et al., 1999; Van den Ende, 2013). However, the synthesis of the small amount of fructan present in banana flesh could be a result of the inherent fructosyl transferase activity of acid invertase (Henderson et al., 1959; Cruz-Cárdenas et al., 2015). Indeed, purified acid invertases can synthesize fructans *in vitro*. This synthesis is favored by high concentrations of sucrose and low amounts of acid invertase, because at higher concentrations of invertase, sucrose and fructans are rapidly degraded by invertase (Pollock et al., 1989; Cairns and Ashton, 1991; Kingston-Smith et al., 1999). In ripe peach flesh, the content of vacuolar sucrose is likely to be high and acid invertase activity low (Moriguchi et al., 1991; Vizzotto et al., 1996; Desnoues et al., 2018), and this would favor the synthesis of fructans as a consequence of the inherent transferase activity of vacuolar acid invertase. Further support for this view in peach comes from studies of the acid invertase gene families. Fructan accumulating species contain further copies of genes that are highly homologous to vacuolar acid invertase, and these encode the fructosyl transferase enzymes used in fructan synthesis (Van den Ende, 2013). No extra copies of vacuolar acid invertase genes were detected in peach (Vimolmangkang et al., 2016), and this suggests that it does not contain genes that encode specialized fructosyl transferases. Similarly, Arabidopsis does not contain fructosyl transferase genes, and the simplest explanation for the presence of small amounts of fructan in this plant is that it they are synthesized by vacuolar acid invertase (Van den Ende, 2013).

## Functions of Non-structural Soluble Carbohydrates in the Flesh

In addition to their role in enticing animals to disperse the fruits and hence their seeds, sugars play several vital roles in the metabolism of the flesh of stone fruits. These functions include providing substrate for metabolism and acting as a major osmoticum used in turgor regulation. Further, the contents of sugars in a tissue can be sensed, and consequently bring about changes in both metabolism and development; and a striking example of this is the induction of fructan biosynthesis in the leaves of many temperate grasses by conditions that increase the sucrose content of the leaf (Pollock et al., 1989; Kingston-Smith et al., 1999).

### Non-structural Soluble Carbohydrates Are the Major Metabolic Substrate Used in Stone Fruit Flesh

Imported sugars provide the bulk of the carbon skeletons used in the synthesis of the non-nitrogenous organic constituents of the flesh (Pavel and DeJong, 1993a; Famiani et al., 2016). In addition, sugars usually provide the bulk of the substrate utilized by the Krebs cycle and respiration to provide NADH and ATP (Famiani et al., 2016). During the growth of whole peach fruits, including their enclosed seed, the proportion of sugars used by respiration is about 20% (Pavel and DeJong, 1993a). For the flesh during ripening, the proportion is likely to be lower, for example, in grape flesh during ripening about 9–14% of sugars present in the flesh are used in respiration (Famiani et al., 2014). In ripe peach flesh, 70–90% of dry matter consists of soluble sugars so roughly 10–30% of the sugars not used in respiration are used in the production of compounds other than soluble sugars. So taking average values for these would mean that about 65% of the soluble sugars imported into the fruit stay as soluble sugars.

### Non-structural Soluble Carbohydrates and Turgor Regulation

Large amounts of sugars are accumulated in the ripening flesh of stone fruits in which they are a predominant osmoticum. Thus, they have a pivotal influence on cell turgor pressure (Winkler and Knoche, 2018). Turgor pressure influences several processes that occur during the growth of the flesh of stone fruits, and these include cell expansion, the import of materials and fruit softening (Wächter et al., 2003; Wada et al., 2008). Sugars increase the turgor pressure of expanding cells and can facilitate cell expansion (Pritchard, 1994). A feature of expanding cells is often a high ratio of (glucose + fructose): sucrose, and these hexoses are often produced from sucrose by acid invertase (Ricardo and ap Rees, 1970; Sturm, 1999). The flesh of stone fruits until stage III of development usually contains a lower content of total sugars and a high ratio of (glucose + fructose): sucrose, and it is possible that this high ratio also facilitates cell expansion at this stage of development when total sugar content is lower and turgor pressure is high. Further, in order for fruits to soften during ripening it is necessary that the parenchyma cells of the flesh do not have a too high turgor pressure, and a suitable concentration of sugars in the apoplast is required to reach a lower turgor pressure (Wada et al., 2008). In a range of tissues including ripening fruit one mechanism that appears to be important in maintaining a suitable concentration of sugars in the apoplast is the hydrolysis of sucrose to glucose plus fructose by cell wall invertase (Wada et al., 2008). Turgor pressure also influences the import of materials via the phloem, and a high turgor pressure inhibits symplastic flow into the tissue (Andersen et al., 2002; Gould et al., 2013). It has been hypothesized for decades that cell wall invertase could play a role in apoplastic phloem unloading, and it was suggested that it could do this by increasing the sucrose concentration gradient between the sites of unloading and sink cells and hence increase the rate of diffusion (Sturm, 1999; Tang et al., 1999). One alternative explanation is that cell wall acid invertase increases the solute concentration in the apoplast by hydrolysing sucrose, which reduces the turgor pressure of the sink cells and facilitates symplastic flow from the phloem. In stone fruits, such as plum, it appears that apoplastic movement via diffusion makes only a very small contribution to apoplastic transport of sugars, and further, there is evidence that phloem unloading can rapidly switch between apoplastic and symplastic (Grappadelli et al., 2019). Thus, it is possible that in stone fruit flesh the relative actions of vacuolar and cell wall invertase can contribute to altering turgor pressure that modifies the contributions of the symplastic and apoplastic pathways to post-phloem transport. Studies on the distribution of cell wall invertase in developing grape berries and their seed are consistent with this view (Walker et al., 1999; Famiani et al., 2000). In the seed coat of developing grape seeds, a specialized tissue called the palisade layer functions to distribute imported assimilates from the phloem to the developing storage tissues. The cells of the palisade layer are connected by numerous plasmodesmata which are thought to facilitate symplastic movement of unloaded assimilates, Further, cell wall invertase is particularly abundant in the palisade layer (Walker et al., 1999; Famiani et al., 2000), Thus, cell wall invertase could potentially function in altering the turgor pressure of the palisade cells, and thus function in regulating symplastic phloem unloading. A feature of many sink tissues is the presence of enzymes that degrade sucrose (invertases and SuSy) together with enzymes involved in sucrose synthesis (SPS and potentially SuSy), and in some of these tissues a cycle of sucrose synthesis and breakdown (termed sucrose cycling) occurs. One function of this cycle could be in sucrose breakdown and re-synthesis associated with turgor regulation (Rossouw et al., 2010; Li et al., 2012, 2016, 2018), and in the flesh of stone fruits it is possible that one function of SPS (and potentially certain forms of SuSy) is in this process.

## Conclusion

From the foregoing, it is clear that the work of a large number of people over several decades has resulted in an impressive understanding of non-structural carbohydrate metabolism in the flesh of stone fruits. What is also apparent is that it is an extremely complex subject, and that there are many intriguing aspects that require further study. These include the subcellular compartmentation of sugars, the distribution of different enzymes between the various tissues of the flesh, the potential cycle of sucrose breakdown and re-synthesis and its functions and the roles of sugars in turgor regulation together with the contribution of enzymes such as acid invertase to this process. In this regard, as stressed by Shiratake and Suzuki (2016), omic studies will, without doubt, contribute to our further understanding of these aspects and other aspects of the metabolism of the flesh of stone fruits (Shiratake and Suzuki, 2016). In this context, important insights can be obtained by integrative omics approaches which combine genomics, transcriptomic, proteomic and metabolomic analyses, in a system biology view, as used in the identification of new candidate genes controlling peach fruit aroma volatiles (Sánchez et al., 2013); or for evaluating the impact of post-harvest treatments on the level of peach fruit polyphenols and related genes (Santin et al., 2019). Further, studies using plants that have altered amounts of either enzymes allied to the sucrose cycle or sugar transporters will provide valuable insights into sugar metabolism in stone fruits as they have done in apple (Li et al., 2018; Wang et al., 2020).

## Author Contributions

All authors have contributed significantly to the work and approved it for publication. However, RW and FF had a major role in the design and writing of the article.

## Conflict of Interest

The authors declare that the research was conducted in the absence of any commercial or financial relationships that could be construed as a potential conflict of interest.
